# Beyond Tradition: An Integrated Toxicological, Ecological, and Public Health Perspective on Aristolochic Acids

**DOI:** 10.1002/jat.70125

**Published:** 2026-03-26

**Authors:** Victor Ventura de Souza, Lara Rodrigues De Andrade, Tatiana da Silva Souza

**Affiliations:** ^1^ Postgraduate Program in Genetics and Breeding, Center for Agricultural Sciences Federal University of Espírito Santo Alegre ES Brazil

**Keywords:** *Aristolochia*, carcinogenesis, nephrotoxicity, phytotherapy, toxicology

## Abstract

*Aristolochia* species have long been used in traditional medicine for their presumed anti‐inflammatory, analgesic and antimicrobial properties. However, extensive toxicological and epidemiological evidence now demonstrates that these plants contain aristolochic acids (AAs) I and II, highly potent nephrotoxic, genotoxic, and carcinogenic compounds. This review integrates findings from experimental models, clinical investigations, and environmental monitoring, emphasizing the persistence, mobility, and bioaccumulation of AAs in ecosystems and food chains. Compelling epidemiological data show that exposure to AAs is strongly associated with Balkan endemic nephropathy (BEN) and upper urinary tract carcinoma (UUC), conditions that exhibit some of the highest rates worldwide in regions of sustained environmental contamination. In Taiwan, UUC incidence is globally unmatched and closely linked to chronic ingestion of AA‐containing herbal preparations, while in Balkan endemic areas, 30%–45% of individuals affected by BEN develop UUC. In these same regions, AA‐derived DNA adducts are detected in the vast majority of exposed populations, serving as highly specific biomarkers of internal dose and demonstrating long‐term mutagenic persistence. Environmental exposure levels further support these associations, with AAs detected in contaminated soils, wheat, and corn grains, and a variety of vegetables grown in endemic villages. Root crops in particular accumulate AAs from soil reservoirs influenced by pH‐dependent solubility and hydrophobicity, while groundwater in affected areas contains AAs concentrations in the ng/L range, revealing an additional exposure pathway through drinking water. Together, these data illustrate a complex exposure landscape that results in preferential accumulation of AAs in renal, hepatic and urothelial tissues, leading to the formation of persistent DNA adducts, characteristic A:T → T:A transversions and subsequent malignant transformation. Despite the clarity of these risks, regulatory responses remain inconsistent across regions, allowing ongoing human exposure through traditional herbal remedies, contaminated food chains, and environmental reservoirs. This review identifies critical research gaps, including the need to better understand chronic low‐dose exposure, interactions with individual genetic susceptibility and the development of more sensitive biomarkers capable of detecting both environmental and clinical exposure. Given the unequivocal risks associated with AAs, harmonized global regulation, strengthened toxicovigilance, and targeted public health education are urgently needed. The replacement of *Aristolochia*‐based preparations with scientifically validated and safer therapeutic alternatives remains essential to reduce the growing global burden of AAs‐related renal and urothelial diseases.

## Abstract 2

*Aristolochia* species, used traditionally for anti‐inflammatory and analgesic effects, contain aristolochic acids (AAs) I and II potent nephrotoxic, genotoxic, and carcinogenic compounds. This review integrates in vitro, in vivo, and clinical evidence, highlighting AAs' environmental persistence, bioaccumulation in food chains, and accumulation in renal/hepatic tissues, inducing DNA adducts, apoptosis, and malignancies. Epidemiological links to Balkan nephropathy and urinary tract carcinomas underscore global health risks. Fragmented regulations exacerbate vulnerabilities. We advocate harmonized policies, toxicovigilance, and safer herbal alternatives to mitigate AA‐related diseases.

## 1 Introduction

**FIGURE 1:**
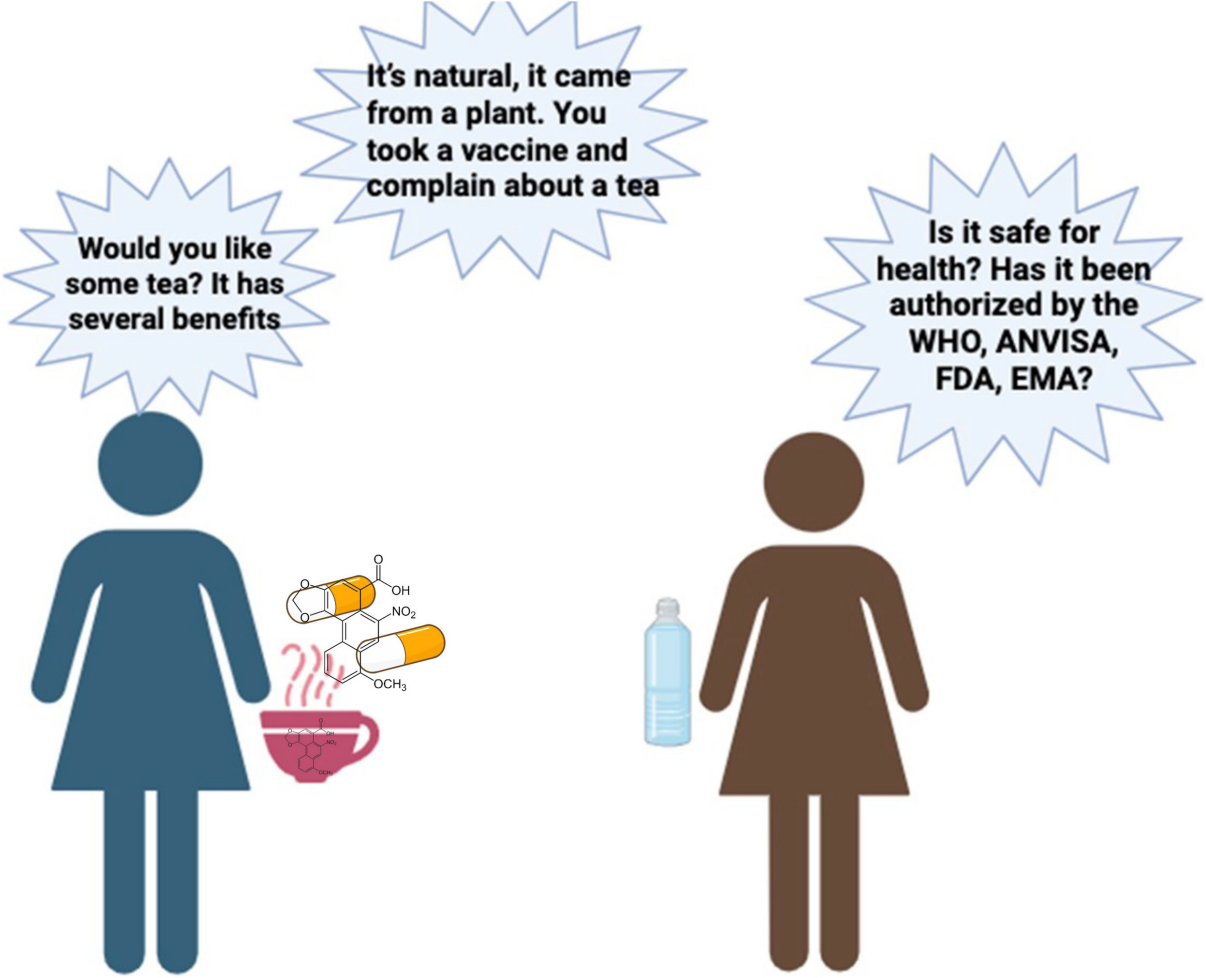
The everyday debate on the benefits and risks of natural products shows that, while popular tradition emphasizes the supposed advantages of herbal teas and other plant‐based preparations, the discussion on safety reinforces the need for scientific validation and regulatory assessment by institutions such as WHO, ANVISA, FDA, and EMA, revealing the persistent tension between empirical practices and contemporary public health standards. Aristolochic acids are not limited to infusions or decoctions prepared from *Aristolochia* leaves, since exposure can also occur through root extracts compressed into pills, which broadens the routes of consumption and makes it essential to represent both forms in the illustration. Another relevant aspect is the frequent botanical misidentification in traditional herbal medicine, a situation that may lead to accidental intoxication when morphologically similar species are confused during harvesting or commercialization, and this issue may also be present in various natural products marketed for weight loss.

The use of plants for medicinal purposes, whether for the treatment, cure, or prevention of diseases, represents one of humanity's oldest healing practices. According to the World Health Organization (WHO), approximately 80% of the global population has relied, or continues to rely, on herbal remedies for alleviating pain or unpleasant symptoms, with at least 30% of these applications occurring under medical supervision (Vendrúscolo and Mentz 2006). Medicinal plants are especially important in many communities due to their widespread accessibility and low cost (Lins and Medeiros 2015). Nevertheless, the traditional use of these plants often occurs without systematic safety assessment, clinical trials, or rigorous approval by governmental agencies, as illustrated in Figure 1.

Among these traditional remedies, teas and preparations derived from plants of the *Aristolochia* have been widely used for their recognized effectiveness in relieving pain caused by snake bites and in treating sexual and gastrointestinal disorders. Other reported applications include tonic and antiseptic properties, as well as effects on the central nervous system (Michl et al. 2013). Such medicinal benefits are attributed to a diverse array of bioactive compounds, including biflavonoids, chalcone‐flavones, tetraflavonoids, lignans, terpenes, diterpenes, sesquiterpenoids, alkaloids, alkamides, and anthraquinones (Chauhan et al. 2024).

However, despite their popularity, numerous studies (Zhang, Wang, Wang, et al. 2022; Tian et al. 2023; Wang, Li, et al. 2024; Cui et al. 2025; Kwak and Chan 2024) have reported that certain alkaloids found in *Aristolochia*, particularly AAI and AAII, share a characteristic nitrophenanthrene carboxylic acid backbone. The principal structural difference lies in the presence of an additional methoxy (—OCH₃) group in AAI, which is absent in AAII (Xing et al. 2012). This seemingly subtle distinction can significantly affect physicochemical properties such as lipophilicity and molecular reactivity, which in turn may influence metabolic activation and DNA‐binding affinity. These structural attributes are closely linked to biological activity: both AAI and AAII have been repeatedly shown to possess toxic and carcinogenic effects in a variety of experimental systems (Wan et al. 2021; Kwak et al. 2025). Despite the long‐standing and widespread use of *Aristolochia* species in folk medicine, a growing body of toxicological evidence now raises serious concerns regarding their safety. Increasingly robust studies have demonstrated that AAs are potently nephrotoxic, mutagenic, and carcinogenic, with the capacity to impair renal function and induce urothelial cancers, among other disorders (Anger et al. 2020; Chen et al. 2022; Luo et al. 2022; Das, Gupta and Roy,  2022; Zhang, Chan, and Chan 2022; Chen et al. 2024; Zhang et al. 2024; Cui et al. 2025; Yang et al. 2025). This evolving scientific consensus exposes a significant disconnect between traditional herbal use and contemporary pharmacological knowledge.

AAs contamination represents a central milestone in the history of clinical toxicology and is a clear example of how traditional practices can result in adverse effects when associated with botanical misidentification or the lack of rigorous phytochemical control. Aristolochic acid nephropathy, characterized by progressive interstitial fibrosis and a high risk of urothelial carcinoma, became widely recognized after the outbreak that occurred in Belgium in the early 1990s, when herbal preparations used for slimming purposes inadvertently contained *Aristolochia* species instead of *Stefania tetrandra*. These cases rapidly progressed to renal failure and revealed the carcinogenic potential of the compound, prompting an extensive expansion of research on its underlying pathogenic mechanisms (Pena et al. 1996; Ioannis et al. 2024). Endemic situations, such as Balkan endemic nephropathy, reinforced this evidence by demonstrating that the continued ingestion of flour contaminated with *Aristolochia* seeds can lead to severe tubulointerstitial renal lesions and a marked increase in upper urinary tract cancer (Chan et al. 2016). Clinical manifestations include hematuria, renal fibrosis, and progression to end‐stage renal disease, associated with molecular mechanisms marked by TP53 mutations, mitochondrial DNA damage, and a persistent inflammatory process, which reinforce the need for early diagnosis and continuous surveillance (Yun et al. 2015).

Furthermore, the largely unregulated use of *Aristolochia*, based products remains a global public health concern, especially in regions where traditional medicine is widely practiced but regulatory oversight is limited or lacking. Disparities in preparation methods, dosages, and plant species can further influence the extent of toxicity, while many traditional applications do not systematically account for these variables. Additionally, underreporting and variation in healthcare infrastructure across countries may obscure the true scale of health risks associated with these plants, thus complicating efforts to develop effective policies and public health interventions. While some studies have proposed potential therapeutic applications for non‐alkaloid constituents of *Aristolochia*, the overwhelming weight of toxicological evidence for AAs emphasizes the urgency for regulated use and comprehensive public awareness. Therefore, it is critical to reconcile ethnobotanical knowledge with rigorous toxicological evaluation to ensure that potential benefits do not come at the cost of significant or irreversible health risks.

This review is organized as follows: we begin by detailing the occurrence and pharmacological characteristics of AAs and their derivatives. Next, we critically examine their toxic, genotoxic, and mutagenic effects across various biological models, with special attention to both mechanistic insights and experimental evidence. We conclude with a discussion of current regulatory challenges, gaps in epidemiological monitoring, and prospects for future research and safer clinical use.

## 2 Effects of Plants of the Genus *Aristolochia* and Aristolochic I (AAI) and II (AAII) on the Test Organisms

### 2.1 Salmonella

Research involving AAs and their mutagenic activity has received considerable attention in scientific literature, particularly through the use of *Salmonella typhimurium* strains in the Ames test, which is internationally regarded as a reference assay for mutagenic risk assessment of environmental and pharmaceutical compounds. AAs, naturally occurring in species of the genera *Aristolochia* and *Asarum*, are characterized by their nephrotoxic and carcinogenic properties and have been associated with severe diseases such as aristolochic acid nephropathy and various types of cancer (Cui et al. 2025; Yang et al. 2025). According to Padhy (2021), the TA98 and TA100 strains exhibit high sensitivity in detecting mutagenicity in plant extracts, and a significant proportion of *Aristolochia indica* samples showed positive results even in the absence of metabolic activation.

**FIGURE 2:**
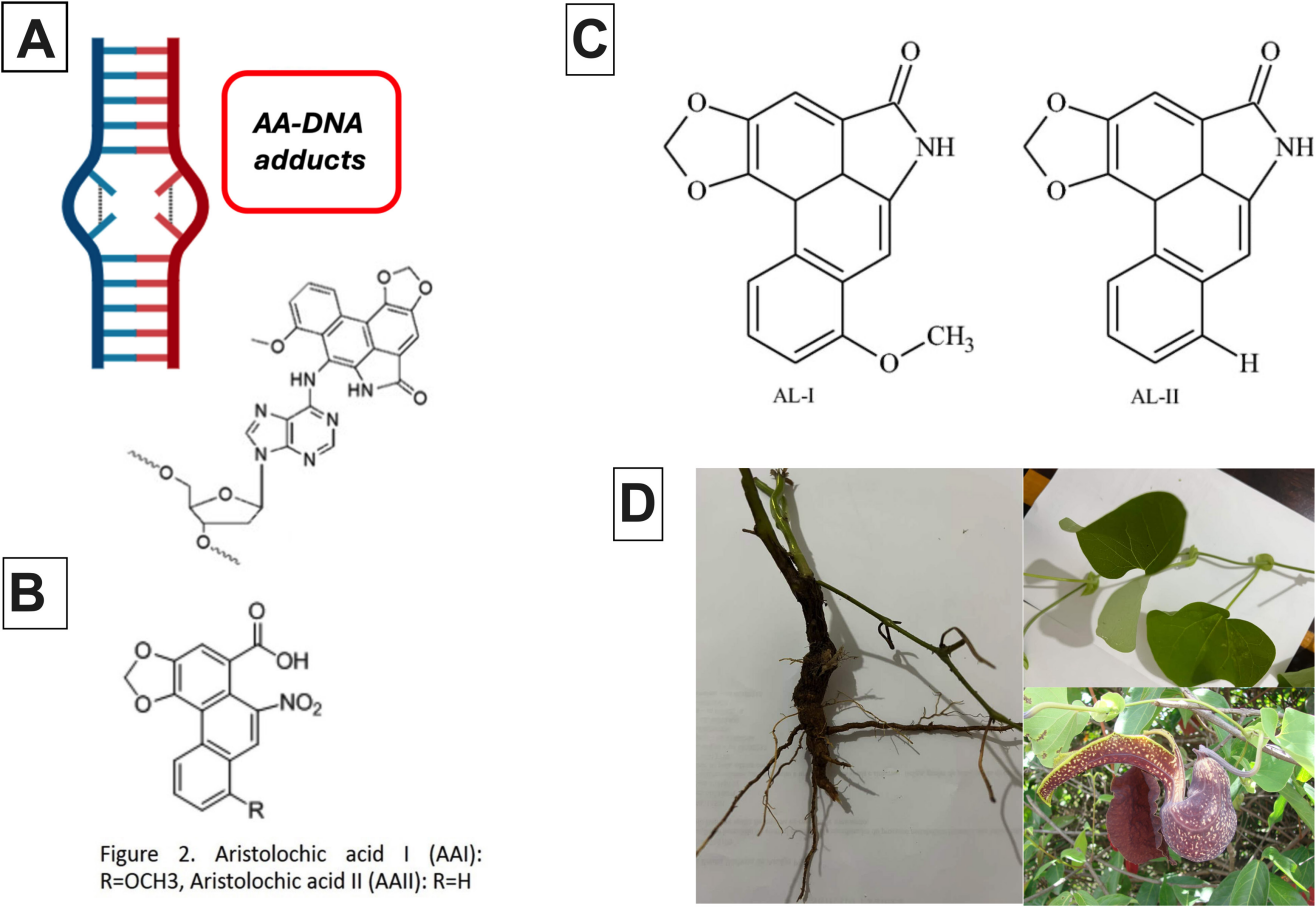
Panel a shows the formation of AA–DNA adducts, resulting from the reductive bioactivation of aristolochic acid into highly reactive intermediates that covalently bind to adenine and guanine bases, producing persistent adducts that represent the typical molecular signature of the genotoxic toxicity of AAs. Panel b presents the chemical structures of aristolochic acid I and II, which share the characteristic nitrophenanthrene carboxylic skeleton, differing in the presence of a methoxy group in AAI, absent in AAII, a structural difference that influences their physicochemical properties, metabolic activation, and DNA binding affinity. Panel c presents the structures of aristolactam I and II, metabolites formed after the reduction of the nitro group of AAs; the structural difference between them stems mainly from the position and pattern of substitution in the aromatic rings, which modifies their electronic and stereochemical properties and affects both stability and the ability to form additional adducts with biomolecules. Panel d *Aristolochia brasiliensis*: native plant from Brazil, found across several biomes including Caatinga, Cerrado, Amazon rainforest, Atlantic Forest, and Pantanal, as well as in several provinces of Argentina. Traditionally used in folk medicine for its purported therapeutic properties.

A particularly relevant aspect in assessing AA‐associated risks is their ability to form stable DNA adducts, notably 7‐(deoxyadenosine‐N6‐yl)aristolactam I, a persistent lesion frequently associated with poor prognosis in hepatic cancer cases and considered a potential initiator of tumorigenesis in mammals (Wang, Zhang, et al. 2022; Wang, Bai, et al. 2022) (Figure 2a). Positive outcomes in the Ames test are often strongly correlated with genotoxic effects in mammalian models. Further studies such as micronucleus and comet assays, as well as analyses of DNA adduct formation in mammalian cells are essential for extrapolating these findings to carcinogenic risk in humans. The integration of bacterial mutagenicity assays with cellular and clinical investigations enhances the understanding of these toxicological hazards and underscores the importance of continued investment in research, regulation, and public awareness to prevent diseases associated with AAs exposure.

### 2.2 Invertebrates

The effects described by Frei et al. (1985) in *Drosophila melanogaster* included high rates of somatic recombination, sister chromatid exchange, chromosomal breaks, loss of sex chromosomes in females, yellow wing spots, and sex‐linked lethal recessivity. These alterations indicated that AAs act directly on DNA integrity and interfere with chromosomal segregation mechanisms. Such genotoxic events reflect both structural and functional chromosomal damage, demonstrating that exposure to AAs can induce mutations, genetic rearrangements, and chromosomal loss. Consequently, distinct phenotypic alterations and sex‐linked lethal effects were observed.

According to Kauts et al. (2024), the occurrence of sister chromatid exchange and somatic recombination suggests increased genomic instability, whereas chromosomal breaks and sex chromosome loss indicate that these compounds may compromise cellular and reproductive viability. Furthermore, Gompel et al. (2005) reported that the appearance of yellow wing spots serves as a visible marker of genetic damage, reinforcing the mutagenic potential of AAs. These findings highlight the importance of studying AAs as genotoxic agents in experimental models and emphasize their potential risks to more complex organisms, including humans.

In studies involving invertebrates, Krishnappa and Elumalai (2012) investigated crude alcoholic extracts from the leaves of *Aristolochia bracteata* and reported that these compounds completely inhibited egg hatching in several mosquito species, including *Aedes aegypti* , *Anopheles stephensi*, and *Culex quinquefasciatus* . Similar results were observed by Pradeepa et al. (2015), indicating that *Aristolochia* extracts possess insecticidal properties relevant to the control of tropical disease vectors. These findings confirm that *Aristolochia* contains biologically active compounds that should be thoroughly isolated, characterized, and evaluated in controlled toxicological studies to ensure their safe and effective application in vector management.

Overall, studies involving invertebrate models, particularly *Drosophila* and mosquito vectors, play a crucial role both in assessing the toxicological risks of AAs and in exploring new strategies for the biological control of medically important arthropods. These models enable rapid and efficient elucidation of toxicity mechanisms and serve as a scientific foundation for the development of sustainable and rational bioproducts aimed at improving public health outcomes.

### 2.3 Fish

AAs are recognized as highly toxic substances, and recent studies using zebrafish ( *Danio rerio* ) have revealed a wide range of adverse effects during both embryonic and larval development. Embryos exposed to varying concentrations of these compounds exhibited cardiac malformations, heart hypertrophy, absence of the endocardium, and disorganized and fragile cardiac fibers (Ding and Chen 2012; Zhang et al. 2025). These structural abnormalities were associated with reduced heart rate, pericardial edema, delayed somatic growth, and slower yolk absorption, demonstrating that AAs compromise both the morphology and the functionality of the cardiovascular system.

From an immunological perspective, AAI displayed marked immunotoxic activity (Figure 2b). Early exposure to AAI significantly reduced the number of macrophages, neutrophils, and T lymphocytes in zebrafish, impairing immune cell migration to injury sites and weakening the overall immune response (Zhang et al. 2025). This effect was primarily linked to oxidative stress induction and the activation of the p53 signaling pathway, leading to apoptosis of immune cells. Antioxidant interventions, such as the administration of fullerene, were able to inhibit pathway activation, prevent cell death, and restore immune cell populations, demonstrating the central role of oxidative stress in AAI‐induced immunotoxicity (Zhang et al. 2025).

Regarding renal function, prolonged exposure of zebrafish larvae to AAI caused evident renal lesions, including loss of glomerular barrier integrity and alterations in the proximal tubules (Wang et al. 2020). In telomerase‐deficient models, abnormal collagen deposition was observed, suggesting that the regenerative capacity of zebrafish can, to some extent, limit AA‐induced fibrosis. The maintenance of tissue homeostasis through continuous renal cell renewal appeared to be crucial in preventing the accumulation of chronic damage (Marro et al. 2016; Bates et al. 2018).

Overall, transcriptomic analyses revealed the activation of multiple molecular mechanisms associated with apoptosis, oxidative stress, and fibrogenesis, including pathways such as PERK/ATF4/CHOP, ATM/Chk2/p53, caspase/Bax/Bcl‐2, TGF/Smad/ERK, and PI3K/Akt (Chen et al. 2023). While the identification of these molecular routes is essential to elucidate the mechanisms of toxicity, immunohistochemical approaches are recommended to confirm protein expression, emphasizing that toxicity arises from the activation of pro‐apoptotic and inflammatory responses that culminate in the systemic impairment of vital organs.

Although zebrafish constitute a valuable model for studying the systemic toxicity and molecular mechanisms of AAs mainly due to their genetic and physiological similarity to humans, ease of manipulation, and low maintenance cost, it is crucial to acknowledge their limitations (Maru et al. 2022). These include differences in drug metabolism, the absence of certain renal structures typical of humans, and the limited translation of larval findings to post‐larval or adult stages (Madivalar et al. 2024). Consequently, additional studies employing other animal models and human cell cultures remain essential for validating the observations and for a more comprehensive understanding of the potential human health risks associated with AAs exposure.

In summary, findings obtained using zebrafish suggest that AAs pose a significant health risk, capable of inducing cardiac malformations, immunosuppression, and renal damage during early developmental stages. However, further research is still required to evaluate the chronic impacts of AAs on adult zebrafish. Such investigations are fundamental for establishing preventive strategies and promoting the safe use of medicinal plants containing these compounds.

### 2.4 Mammals

This section encompasses studies performed in vivo and in vitro on mammalian models.

#### 2.4.1 Rabbits

Experimental investigations using rabbits have provided detailed insights into the pharmacokinetic behavior and renal toxicity of AAs in this model system. Pharmacokinetic analyses reported by Chen et al. (2007) demonstrated that, following intravenous administration of 0.25–2.0 mg/kg, the principal components AAI and AAII undergo relatively rapid elimination, typically completed within 3 h. Under repeated dosing, however, the compounds exhibit nonlinear accumulation, reflected by a prolonged half‐life and reduced plasma clearance, thereby reinforcing the risk of cumulative toxicity. Histopathological evaluations revealed that renal injury is predominantly localized to the proximal tubules, characterized by progressive epithelial atrophy and the formation of hyaline casts at higher doses, while glomerular structures remain preserved. The severity of these lesions shows a direct correlation with dose and duration of exposure, confirming that AA‐induced nephrotoxicity follows a cumulative and progressive pattern.

According to Podolan et al. (2022) and Wancket et al. (2022), the rabbit represents an appropriate experimental model due to its anatomical and physiological similarities to humans, particularly in the renal and cardiovascular systems, as well as its suitable body size and high sensitivity to toxic agents. These features make rabbits useful for controlled and clinically relevant toxicological assessments.

The findings obtained in rabbits corroborate results from zebrafish and other animal models, highlighting the multifactorial nature of AAs toxicity, which affects multiple organs and systems. In particular, the severe nephrotoxicity induced by AAs seems to result from a combination of direct tubular injury, persistent chronic inflammation, and induction of fibrosis and carcinogenesis. The relative integrity of the glomeruli suggests the proximal tubules as the main target, likely due to their high expression of metabolic enzymes capable of converting AAs into reactive intermediates. The presence of anemia and polychromatophilic leukocytosis, in association with renal alterations, indicates that toxicity extends beyond renal tissue, affecting both hematopoietic and immune systems and reflecting the systemic complexity of AA‐induced damage.

Overall, the robustness of data obtained in rabbits underscores their relevance for understanding the progression and multifactorial mechanisms of AA‐induced toxicity. However, the range of affected organs reinforces the necessity of comparative studies involving other animal species and human models to establish early detection strategies and effective interventions against AA‐induced nephrotoxicity.

#### 2.4.2 Effects of Aristolochic Acids in In Vitro Cell Cultures

**FIGURE 3:**
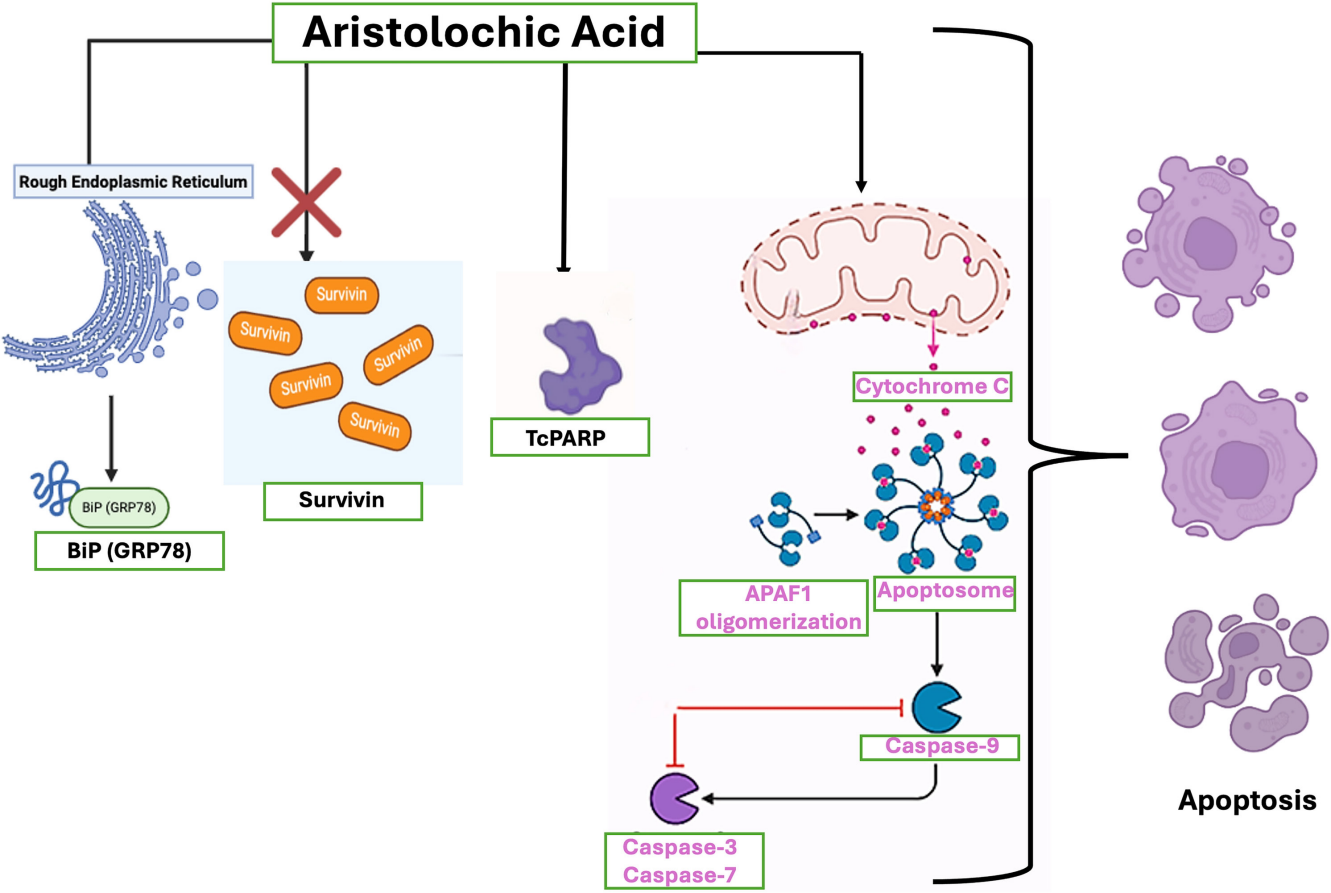
Schematic representation of apoptosis induced by aristolochic acids (AAs). AAs promote the synthesis of pro‐apoptotic proteins such as caspase 9 (cysteine‐aspartic protease 9), caspase 3 (cysteine‐aspartic protease 3), and caspase 7 (cysteine‐aspartic protease 7), which act in the execution phase of apoptosis, as well as PARP (Poly[ADP‐ribose] polymerase), involved in DNA damage detection and repair. Simultaneously, anti‐apoptotic proteins such as Survivin (BIRC5, Baculoviral IAP Repeat Containing 5) are inhibited, favoring cell death. In addition, AAs induce stress in the rough endoplasmic reticulum, leading to the release of GRP78 (BiP, Binding Immunoglobulin Protein), which signals cellular stress, and mitochondrial stress resulting in the release of APAF‐1 (Apoptotic Protease Activating Factor 1) and cytochrome C, both essential for apoptosome formation. Note in the figure the release of cytochrome C from mitochondria and its association with APAF‐1 forming the apoptosome, which triggers the caspase cascade culminating in the final product: apoptosis.

In vitro studies using renal epithelial cell lines such as LLC‐PK1 and NRK‐52E have demonstrated that AAs, particularly AAI, exert pronounced cytotoxic effects on proximal tubular cells. Balachandran et al. (2005) reported that AAs induce apoptosis mainly through the activation of Caspases 3 and 7. Similarly, Li et al. (2006) observed that after 24 h of exposure to AAI, some cells exhibited DNA damage, cell lysis, and G_2_/M cell‐cycle arrest, independent of wild‐type p53 activity. Additional investigations, such as those by Yang, Dou, et al. (2011), described loss of plasma membrane integrity and release of cytochrome c from mitochondria. This process was accompanied by increased expression of the pro‐apoptotic protein Bax and decreased expression of the anti‐apoptotic protein Bcl‐2, underscoring the central role of the mitochondrial pathway in apoptosis induction, as illustrated in Figure 3.

Supporting these findings, Shi and Feng (2011) and Liu et al. (2020) confirmed that AAI directly impaired mitochondrial function in NRK‐52E cells, leading to increased levels of reactive oxygen species (ROS), reduced mitochondrial membrane potential, decreased mitochondrial DNA copy number, and diminished ATP production. The resulting apoptosis occurred in a dose‐ and time‐dependent manner. Interestingly, treatment with mitochondrial‐targeted antioxidant peptides such as Szeto‐Schiller 31 attenuated these alterations, demonstrating that mitochondrial dysfunction represents a key mechanism in AA‐induced cytotoxicity.

Beyond renal epithelial toxicity, AAs also exhibited substantial cytotoxicity in endothelial cells. In human cell models, Bastek et al. (2019) demonstrated that AAI formed DNA adducts in human renal proximal tubular epithelial cells (RPTEC/TERT1 and pHKC), independent of NQO1 expression. This finding suggests that multiple enzymatic systems are involved in AAI bioactivation. The fact that DNA adduct formation did not correlate linearly with cell viability highlights the complexity of this toxicological relationship and underscores the need for more sensitive methods to monitor AA‐induced nephrotoxicity in humans.

**FIGURE 4:**
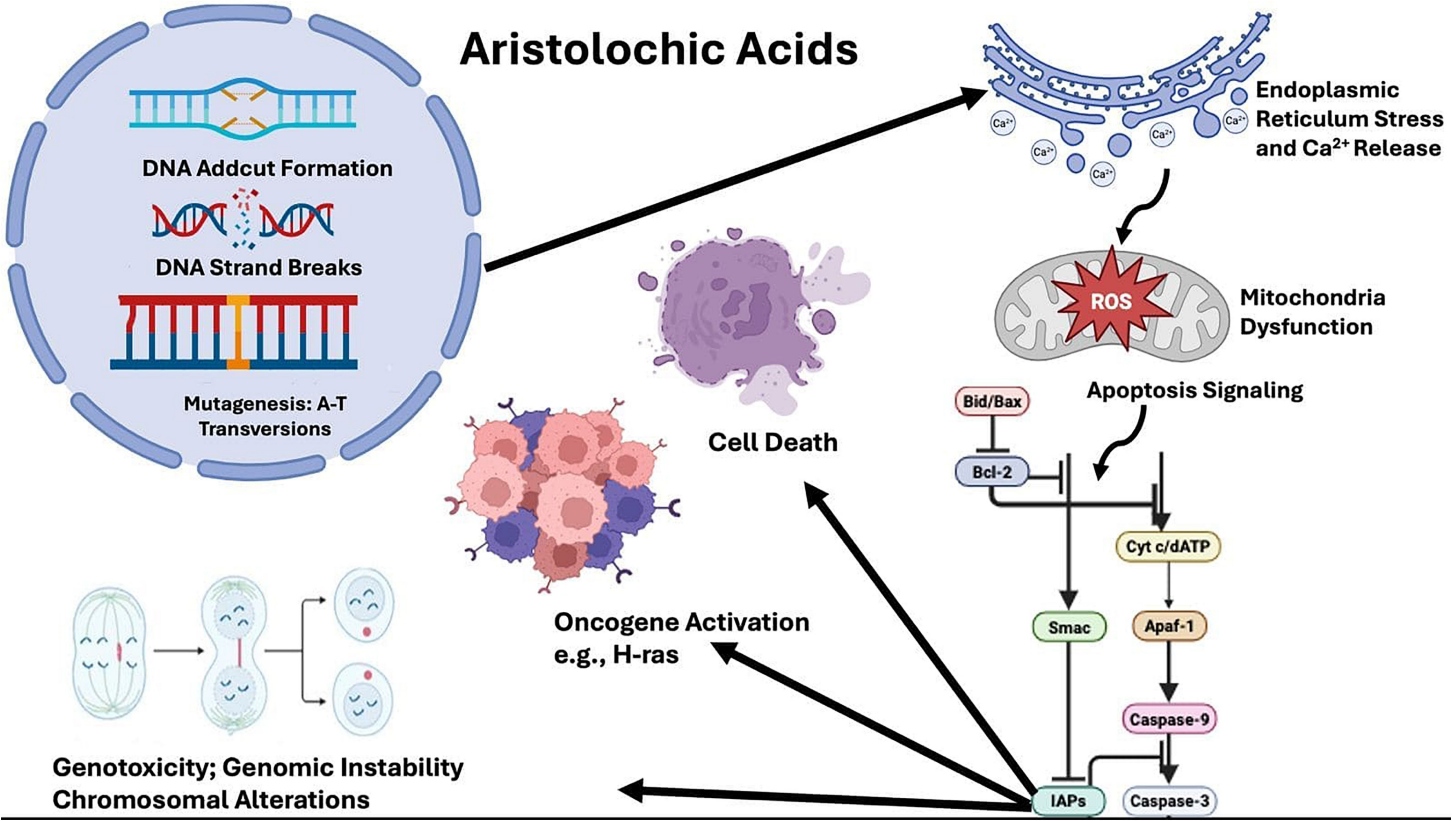
Multifaceted effects of aristolochic acids on cells: exposure leads to DNA adduct formation, which triggers double‐strand breaks, A → T transversion mutations, and chromosomal alterations, promoting genotoxicity and genomic instability. These molecular damages are strongly implicated in the activation of oncogenes such as *H‐ras* and in cell death. Concurrently, endoplasmic reticulum stress with Ca^2+^ release contributes to mitochondrial dysfunction and the generation of reactive oxygen species (ROS), activating apoptotic signaling pathways via Bid/Bax proteins, Bcl‐2, the cytochrome c/dATP complex, Smac, Apaf‐1, and caspases 9 and 3, while inhibitor of apoptosis proteins (IAPs) negatively regulate this process. Altogether, these events culminate in intense cytotoxicity, genotoxicity, and an increased risk of neoplastic transformation, emphasizing the severity of aristolochic acid‐related health hazards.

Major Cellular Mechanisms Involved. Overall, evidence indicates that AA‐induced toxicity in vitro results from the interplay of multiple mechanisms, including mitochondrial dysfunction, oxidative stress, altered calcium homeostasis, imbalance between pro‐ and anti‐apoptotic proteins, induction of autophagy, interference with protein synthesis, and formation of DNA adducts (Upadhyay and Batuman 2022; Lukinich‐Gruia et al. 2024). This complex cellular network suggests that the toxic effects of AAs extend beyond the impairment of a single organ or molecular pathway, leading to broad systemic cellular disturbances that increase the risk of renal injury, fibrosis, and carcinogenesis (Au et al. 2023). These interconnected mechanisms are schematically represented in Figure 4, which illustrates the principal cellular events triggered by AAs (Anger et al. 2020; Luo et al. 2022).

Extrapolating these findings to humans, it is plausible that chronic exposure to low doses of AAs may induce interstitial fibrosis, proximal tubular atrophy, and impairment of renal microcirculation, exacerbating tissue hypoxia and promoting the development of aristolochic acid nephropathy (Kang et al. 2021). Furthermore, the persistent formation of DNA adducts and activation of classical apoptotic pathways may contribute to the emergence of urothelial tumors and potentially to carcinogenesis in other tissues, such as the liver, lungs, and reproductive system, as supported by data from animal models.

#### 2.4.3 Rodents

Nephrotoxicity in Rodents. Nephrotoxicity represents the most characteristic hallmark of AAs in rodent experimental models. Since the earliest investigations, these compounds have shown a strong renal tropism, leading rapidly to both acute and chronic injury (Liu et al. 2003). Various studies have explored different sources and modes of exposure. For instance, the administration of *Aristolochia rotunda* L. extract to rats (Abolhasanzadeh et al. 2023) produced classical pathological alterations, including increased urinary excretion of glucose and protein, dilation of proximal tubules, and extensive mitochondrial damage within renal tissue, all unmistakable indicators of acute tubular injury. Among these compounds, AA‐I has been identified as the primary nephrotoxic agent, targeting proximal tubular epithelial cells preferentially.

This cellular injury arises through interconnected mechanisms: AA‐I promotes the accumulation of ROS, induces mitochondrial DNA depletion, and causes loss of mitochondrial membrane potential. Consequently, ATP synthesis becomes critically impaired, restricting the ability of cells to sustain essential physiological processes and predisposing the kidney to apoptosis and necrosis (Upadhyay and Batuman 2022; Lukinich‐Gruia et al. 2024).

Typically, AA‐induced nephrotoxicity begins with an episode of acute renal injury marked by elevated serum creatinine and blood urea nitrogen, tubular necrosis, interstitial fibrosis, and activation of myofibroblasts. Without timely intervention or withdrawal of exposure, this condition often progresses to chronic kidney disease, characterized by tubular atrophy, collagen deposition, and progressive deterioration of renal excretory and regulatory capacities (Mei et al. 2022; Baudoux et al. 2022; Reinoso et al. 2024). Prolonged exposure to aristolochic acid IIIa has also been shown to induce focal tubular lesions and fibroblast hyperplasia even at sub‐acute doses, suggesting cumulative toxicity and mutagenic potential (Wang, Fan, et al. 2024).

An innovative finding in recent years has been the demonstration of ferroptosis involvement a distinct form of regulated cell death characterized by lipid peroxidation and reduced antioxidant capacity. This process deepens the tubular injury already initiated by mitochondrial dysfunction, adding another dimension to AA‐induced renal damage (Huang et al. 2024).

Another pivotal aspect concerns sex‐related variability in susceptibility. Male mice have been found to be more vulnerable to AA‐induced renal toxicity than females, and orchiectomy exhibited a protective effect, suggesting hormonal participation and possible gene–sex interactions modulating toxicity (Li et al. 2023). Inflammation also plays a critical aggravating role, with AA‐I eliciting robust innate immune responses that further exacerbate renal tissue injury (Upadhyay and Batuman 2022).

Hepatotoxicity and Carcinogenesis. Although AAs have long been associated primarily with nephrotoxicity and urothelial carcinogenesis, recent evidence has revealed broader toxic potential most notably involving the liver, a central organ in xenobiotic metabolism. Studies in mice have provided compelling evidence of hepatic susceptibility. Exposure to AA‐I triggered liver damage whose severity was directly influenced by the animal's age, with adult mice exhibiting considerably greater hepatocellular injury than neonates following chronic administration (Fang et al. 2022). These findings suggest that, when extrapolated to humans, factors such as age, prior hepatic condition, and exposure history may modulate the toxic response to AAs, rendering risk assessment a multifactorial and dynamic challenge.

Concern deepened after the identification of typical AA‐derived DNA adducts such as 7‐(deoxyadenosine‐N6‐yl)aristolactam I in human liver tumors (Wang, Zhang, et al. 2022; Wang, Bai, et al. 2022). The presence of these adducts not only confirms the ability of AAs to reach the liver and induce persistent genotoxic lesions but also supports their potential involvement in hepatic oncogenesis, particularly in tumors with poor clinical outcomes. This evidence is strengthened by data from human liver cell cultures, which revealed high cytotoxicity and low IC_50_ values for compounds such as aristolactam BII and AA‐I (Zhang, Chan, and Chan 2022). DNA damage was also confirmed in comet assays showing extensive strand breaks, thereby establishing a mutagenic scenario conducive to tumor initiation (Geric et al. 2025).

To understand these phenomena, it is necessary to consider the complex biological responses elicited by AA‐I. In hepatocytes, this compound activates pro‐inflammatory pathways such as NF‐κB and STAT3, triggering necrosis, apoptosis, and oxidative stress. In sinusoidal endothelial cells, AA‐I induces inflammation and cell death, jointly compromising hepatic microarchitecture and function (Luo et al. 2022). Moreover, AA‐I negatively modulates the farnesoid X receptor (FXR), a key regulator of bile acid and lipid metabolism, leading to dyslipidemia and chronic inflammation, thereby amplifying metabolic disturbances and hepatic injury (Ma et al. 2024). Collectively, these results indicate that AAs act both as direct inducers of cell death and as mediators of profound metabolic imbalance.

In the context of carcinogenicity, the covalent formation of DNA adducts such as 7‐(deoxyadenosine‐N6‐yl)aristolactam I serves as a molecular fingerprint of exposure and is implicated in chronic mutagenic processes. However, the causal relationship is not absolute. Although the presence of these adducts in human tumors reinforces the potential of AAs as initiators or co‐carcinogens, population analyses reveal that the specific mutational signatures of AAs, which are common in urothelial carcinomas, are less frequent in hepatic cancers (Lim et al. 2022; Li et al. 2024). This suggests that in the liver, the carcinogenic contribution of AAs is context‐dependent, modulated by factors such as genetic background, duration and mode of exposure, and the presence of environmental cofactors.

Nevertheless, the evidence from animal models must be interpreted with caution. Some variants of AAs, such as aristolochic acid IIIa, induce pre‐gastric carcinomas in rodents lesions with limited relevance to humans, since the forestomach is absent in human anatomy. Moreover, even after long‐term exposure, significant histological alterations in the liver are not consistently observed (Wang, Fan, et al. 2024). These findings emphasize that direct extrapolation from rodent models to human clinical contexts is inappropriate; rather, experimental outcomes should serve as guides for future epidemiological and clinical research.

Taken together, the data illustrate the persistent controversies surrounding AA‐induced hepatotoxicity. For centuries, *Aristolochia* species have been used in traditional medicine without consistent reports of hepatic toxicity, suggesting that additional or synergistic factors such as genetic polymorphisms, cumulative accidental dosing, or concurrent exposure to other environmental toxins may contribute to the manifestation of severe outcomes. This presents a complex and nuanced scenario in which experimental evidence undeniably warrants strict regulatory measures, yet premature generalizations could generate unnecessary alarm or disrupt culturally rooted medical practices without sufficient clinical evidence.

Hence, a comprehensive, multidisciplinary approach integrating molecular biology, epidemiology, and ethnopharmacology is essential to elucidate the true magnitude of AA‐related risk. Only through such integration will it be possible to support balanced public health policies, develop effective preventive strategies, and formulate health recommendations that respect both traditional knowledge and modern safety standards.

Effects on Other Organs and Systems: Beyond the Kidney and Liver. Historically, studies of AAs in rodents have focused primarily on nephrotoxicity and hepatic carcinogenesis. However, as scientific understanding has advanced, it has become evident that the toxicity of these compounds extends to multiple organ systems, resulting in a systemic pattern of injury whose full complexity is still being elucidated (Cui et al. 2005). The best‐established example of such extended toxicity occurs in the male reproductive system. Experiments conducted by Kwak and Lee (2015) demonstrated that exposure to AAs caused a significant reduction in testicular weight and a marked decline in spermatogenesis. These alterations were closely associated with the activation of pro‐apoptotic pathways involving ERK1/2, BAX, BAK, and caspases 3 and 9, accompanied by PARP fragmentation and suppression of the anti‐apoptotic protein Bcl‐2. This cascade leads to apoptosis of Sertoli cells, directly impairing sperm production. Although research on other aspects of reproductive function remains limited, the genotoxic and systemic nature of AAs clearly widens the risk of fertility impairment and highlights the need for further studies on potential multigenerational effects.

The damaging potential of AAs is not restricted to reproductive tissues. Pozdzik, Salmon, Debelle, et al. (2008) reported that in the kidneys, in addition to classic tubular necrosis, there was intense infiltration of immunocompetent cells, indicating renal immune activation and local inflammation. Complementary studies by Bhalli et al. (2013) revealed that DNA damage was not confined to renal tissue but also affected the liver and bone marrow, showing that AAs impact hematological and immune systems and have multiple systemic consequences for organismal homeostasis. Emerging evidence also suggests that the nervous system may be susceptible to these compounds. Although direct studies are scarce, the generalized oxidative stress and mitochondrial dysfunction triggered by AAs likely compromise high‐energy‐demand tissues such as neural ones. Given that mitochondrial integrity is crucial for neuronal survival, it is plausible that chronic or continuous exposure could lead to neurological dysfunctions whose underlying mechanisms still require further clarification (Lukinich‐Gruia et al. 2024).

More recently, AA‐induced metabolic disturbances have also been described in the circulatory system. Altered gene expression and proteomic profiles caused by AAs exposure provoke systemic inflammatory responses and elevated oxidative stress, creating a microenvironment conducive to vascular injury and circulatory disorders. Such alterations may promote atherosclerotic formation and impair microcirculation, thereby increasing the risk of cardiovascular events (Anger et al. 2020). The overall picture becomes even more complex when considering the potential involvement of the respiratory system.

Therefore, although traditionally associated with renal and toxicities, AAs clearly exert broad and interconnected effects that span the reproductive, hematological, nervous, circulatory, and respiratory systems. Given this wide spectrum of injury, it is essential to deepen investigations into systemic mechanisms and target organs while reassessing regulatory strategies and public‐health approaches aimed at minimizing risks and better understanding the multiorganic consequences of these compounds.

### 2.5 Molecular Mechanisms and Genomic Instability

The systemic toxicity of AAs is based on complex molecular mechanisms that are widely documented in scientific literature. One of the most striking biochemical features of AAs and *Aristolochia* species is their ability to form DNA adducts (Wan et al. 2021). Reactive AAs metabolites can covalently bind to adenine and guanine bases, distorting the DNA double helix and promoting base transversions, particularly adenine‐to‐thymine (A → T) substitutions (Kathuria et al. 2020). The formation of these DNA adducts occurs after enzymatic bioactivation of AAs, which converts the molecules into electrophilic intermediates capable of reacting directly with nitrogenous bases. These reactive metabolites bind covalently, predominantly to adenine and guanine, generating stable adducts that become fixed within the DNA structure and produce distortions in the double helix (Chen 2020). This structural alteration compromises replication fidelity, leads to errors in nucleotide insertion, and results in base transversions. The persistence of these adducts overwhelms the cellular repair capacity and triggers a progressive accumulation of mutations, promoting genomic instability, functional loss, and an environment conducive to malignant transformation over time (Thakur et al. 2022; Jones et al. 2025).

These mutational events correspond to permanent alterations in DNA that arise after the formation of adducts induced by AAs (Chen 2020). When the structure of DNA is distorted, the cell replicates its genetic material inaccurately, inserting incorrect bases and generating mutations that may activate genes normally kept silent, such as oncogenes, including H‐ras, which, once activated, stimulate excessive cellular proliferation (Mei et al. 2006; Slade et al. 2009; Arlt et al. 2011; Takaidza et al. 2025). At the same time, these mutations may inactivate tumor suppressor genes such as p53, which are responsible for halting the cell cycle, repairing damage, or inducing apoptosis in compromised cells (Zhang et al. 2024). The combination of these two outcomes creates a cellular environment characterized by uncontrolled growth, reduced repair capacity, and a higher likelihood of malignant transformation, underscoring the strong mutagenic and carcinogenic potential of AAs. This genotoxic profile is similar to that of well‐known environmental carcinogens, including aflatoxin B₁ and benzo[a]pyrene, further highlighting the high carcinogenic potential of AAs (Thakur et al. 2022; Wang, Fan, et al. 2024; Hellany et al. 2025; Hartwig et al. 2020).

Continuing this molecular perspective, exposure to AAs has been shown to induce marked genomic instability and activate autophagic pathways (Wang, Fan, et al. 2024). This occurs because AAs trigger a substantial increase in oxidative stress, elevating the production of ROS that damage proteins, lipids, and essential nuclear components. In response to this stress state, the cell activates autophagic pathways as an initial defense mechanism aimed at removing dysfunctional mitochondria and other damaged organelles, thereby maintaining cellular homeostasis as described by Shen et al. (2021). However, although autophagy plays a physiological role in damage control and in the preservation of genomic stability, its persistent or excessive activation induced by AAs can become detrimental. Studies demonstrate that dysregulated autophagy further intensifies ROS production, creating a feedback loop that exacerbates DNA damage and contributes to chromosomal aberrations (Cheng et al. 2021; Cheng et al. 2025). Moreover, core proteins of the autophagic machinery interact directly with DNA repair systems and with cell cycle regulators, and their dysfunction results in failures in repair mechanisms and loss of chromosomal integrity, as noted by Zhang, Chan, and Chan (2022). Thus, exposure to AAs produces a paradoxical condition in which autophagy, initially activated as a protective response, gradually becomes an additional driver of genomic instability, especially when oxidative stress exceeds the cell's capacity to repair and control damage (Luo et al. 2022). The complexity of this process is amplified by the regulatory role of amino acids in the mTORC1 pathway, which normally acts to inhibit autophagy and prevent excessive activation; when this balance is disrupted, the cell becomes more susceptible to the progression of AA‐induced injury (Shen et al. 2021). In this way, the genomic instability resulting from the interaction among oxidative stress, adduct formation, and autophagic activation establishes a cellular scenario in which AAs not only initiate molecular damage but also compromise the systems responsible for containing it, thereby amplifying their toxic and carcinogenic effects over time. In this context, experimental evidence points to a variety of chromosomal aberrations, as observed by Hwang et al. (2012), while Yang, Dou, et al. (2011) documented acute nephropathy accompanied by excessive autophagy in renal tubular cells following exposure to AAI. Moreover, mitochondrial damage intensified by the accumulation of ROS contributes to worsening overall toxicity (Zhou et al. 2013; Bhalli et al. 2013; Jiang et al. 2013). These alterations not only impair cellular viability and function but also increase the risk of malignant transformation due to the accumulation of mutations and the dysregulation of DNA repair pathways.

Another factor that warrants attention is the comparative toxicity between the AAs fractions. As demonstrated by Kwak et al. (2025), AAI exhibits greater toxicity than AAII, likely due to its higher absorption efficiency and tissue bioavailability. This difference is supported by experimental data showing that AAI is absorbed approximately three times more efficiently than AAII in both cultured human cells and intestinal sac experiments, a performance that significantly increases its systemic availability and the amount of metabolites capable of causing damage (Kwak et al. 2025). This intensified absorption allows AAI to reach higher concentrations in tissues, promoting greater DNA adduct formation and higher levels of oxidative stress, which are central mechanisms underlying its nephrotoxic and carcinogenic profile. In addition, the presence of the organic anion transporters OAT1 and OAT3 in the kidney enhances the uptake and accumulation of AAI, further increasing its renal toxicity; notably, inhibition of these transporters reduces compound accumulation and attenuates tubular damage, reinforcing their critical role in tissue distribution (Kwak et al. 2025). The greater toxicity of AAI is also related to its more efficient reductive activation, which intensifies the production of electrophilic metabolites and, consequently, DNA damage when compared with AAII, as shown by Kwak et al. (2025). Moreover, studies indicate that co‐exposure to AAI and AAII can exacerbate the genotoxic effects of AAI because AAII can inhibit its detoxification, prolonging its retention in tissues and favoring adduct formation, as reported by Bárta et al. (2021). Pharmacokinetic differences between the two fractions are also relevant, with AAI showing faster absorption and elimination, whereas traditional processing methods, such as honey‐processing of *Aristolochiae fructus*, have been shown to reduce the absorption of both AAI and AAII and thus decrease their toxicity, as observed by Yuan et al. (2017). In summary, the molecular mechanisms triggered by AAs not only clarify the breadth and diversity of their toxic effects but also reveal an integrated cascade of events that extends far beyond the mere formation of DNA adducts. The convergence of oxidative stress, genomic instability, mitochondrial dysfunction, and dysregulated autophagic activation creates a profoundly vulnerable cellular environment in which multiple defense systems are simultaneously overwhelmed. This complex interplay confers upon AAs a unique capacity to compromise several organs in a parallel and progressive manner, reinforcing their profile as high‐risk compounds whose action is systemic, persistent, and potentially carcinogenic in both experimental contexts and clinical scenarios of inadvertent exposure.

### 2.6 Methodological Challenges and Limitations in Extrapolating the Effects of Aristolochic Acids and *Aristolochia* sp. in Bioindicators to Human Health

The evaluation of AAs toxicity in animal models has contributed to understanding potential risks to human health, and this becomes clearer when the difference between experimental doses and the real exposure levels documented in populations consuming *Aristolochia* species for decades is examined in detail. This contrast is evident in classical studies such as that of Pozdzik, Salmon, Husson, et al. (2008), which administered 10 mg/kg per day of aristolochic acid to rats, a value that exceeds by hundreds of times the concentrations typically ingested by humans. In human populations, as demonstrated by Slade et al. (2009), exposure occurs at low yet continuous levels, generating characteristic mutational signatures that reflect long‐term accumulation. The discrepancy between high laboratory doses and cumulative, low‐intensity human exposure introduces a methodological gap that influences interpretation and shows that toxic responses in rodents tend to emerge only at concentrations that substantially exceed what would be physiologically plausible for humans.

This difference becomes even more evident when considering subchronic studies such as Hwang et al. (2006), which tested extracts containing approximately 0.05 mg/kg per day up to nearly 5 mg/kg per day of AAs, corresponding to dozens or hundreds of times the intake associated with real human use. Even the lowest doses that produced measurable effects in the study, around 0.5 mg/kg per day, remain far above what is estimated for individuals consuming traditional preparations. Similarly, research such as that of Chang et al. (2006), although not publicly detailed in all dosage specifics, follows the pattern of employing elevated concentrations to induce urothelial changes, reinforcing how much experimental protocols have diverged from actual human scenarios.

The issue is further complicated by the absence of standardized extraction methods, which introduces substantial chemical variation. Studies conducted by Arlt et al. (2011), Zhou et al. (2013), and Kwak et al. (2025) show that solvent choice, extraction temperature, and extraction duration deeply affect the final concentration of AAs detected. More polar solvents tend to extract higher concentrations, while higher temperatures can both increase compound release and promote degradation, altering the phytochemical profile. This means that two extracts obtained from the same species can have very different concentrations, directly affecting toxicity evaluation and introducing uncertainty into the interpretation of biological outcomes. The complexity increases when considering, as demonstrated by Zhang et al. (2024) and Yang et al. (2025), that AAs content varies widely between *Aristolochia* species and even within a single species depending on geographic origin, cultivation practices, or harvest season, reinforcing the need for strict phytochemical control.

Translating rodent findings to human biology is further challenged by physiological differences, including metabolic pathways, xenobiotic biotransformation, and the presence of anatomical structures absent in humans. Recent studies, such as those by Wang, Fan, et al. (2024), Wang, Li, et al. (2024), and Tian et al. (2023), have advanced by implementing more precise chemical controls and designing exposure scenarios that better reflect human conditions, yet a substantial distance remains between experimental protocols and actual human exposure patterns. This disconnect is intensified by the lack of regulatory oversight in various regions where *Aristolochia*‐based preparations are still traditionally used, as noted by Das et al. (2022) and complemented by reports such as those of Lestari et al. (2025), indicating a scenario in which populations remain at risk due to insufficient monitoring.

For the scientific field to generate evidence capable of guiding decision‐making and public health recommendations, it is essential for studies to adopt stricter phytochemical characterization, dosing regimens that reflect real patterns of consumption, and methodological harmonization that reduces the distance between laboratory designs and actual human exposure scenarios. Moving in this direction strengthens mechanistic understanding, improves risk assessment, and supports the development of guidelines that respect traditional practices while addressing current health protection needs. The ability of experimental work with AAs to inform clinical practice and public health policy depends directly on these uniform methodological standards, since only when animal exposure models approximate human consumption patterns is it possible to produce reliable evidence to understand the effects on terrestrial organisms in a way that is meaningful for real populations. Rigorous phytochemical monitoring, the establishment of doses aligned with traditional use, and effective international regulatory protocols are therefore essential steps for generating consistent knowledge on AAs toxicity. Such harmonization ultimately enables the formulation of safe and scientifically grounded recommendations that reconcile traditional knowledge with contemporary public health demands.

### 2.7 Humans

Human exposure to AAs occurs mainly through two primary routes: the intentional or accidental consumption of herbal products derived from *Aristolochia* species, and the ingestion of contaminated food in specific regions such as the Balkans and parts of Asia, where the presence of AAs in soil and water can lead to their accumulation in agricultural crops (Cui et al. 2025; Yang, Su, et al. 2011; Stiborová et al. 2016). Documented reports include cases of individuals who unknowingly ingested contaminated teas or herbal preparations, as well as cultural practices involving the use of homemade remedies frequently sold in informal markets without any regulatory control (Nortier et al. 2000; Okhale et al. 2019; Anger et al. 2020; Karanović et al. 2022).

In all these circumstances, the primary toxicological concern lies in the biochemical mechanism of bioactivation, in which hepatic and renal metabolism convert AAs into reactive intermediates through the action of enzymes such as NAD(P)H:quinone oxidoreductase, cytochromes P450 1A1/2, and sulfotransferases. These intermediates form covalent bonds with DNA, generating persistent aristolactam–DNA adducts. This process underlies the characteristic nephrotoxicity of AAs, the *TP53* mutational signature (A:T → T:A transversion) commonly found in urothelial carcinomas, and the high incidence of severe chronic kidney disease and upper urinary tract carcinomas in exposed populations (Schmeiser et al. 2008; Stiborová et al. 2013; Jelaković, Castells, et al. 2015).

The occurrence of urothelial carcinoma in individuals exposed to AAs, as described by Nortier et al. (2000), Jelaković, Lela, et al. (2015) and Wu and Wang (2013), represented a major turning point in understanding the disease, since it demonstrated that toxicity is not limited to renal impairment but also includes a markedly increased risk of malignant transformation throughout the urothelium, involving both the upper urinary tract and the bladder, a pattern highlighted in the synthesis by the IARC (2012). These neoplasms may arise early, even before clear signs of renal insufficiency, as shown by Nortier et al. (2000), or may appear later, including after kidney transplantation or in patients undergoing dialysis, as documented by Roumeguère et al. (2014), which broadens the clinical complexity and requires differentiated strategies for surveillance and follow‐up. This variability is linked to individual differences in metabolic processes capable of activating or detoxifying AAs, modulated by enzymatic polymorphisms discussed by Stiborová et al. (2013), which influence the formation and persistence of DNA adducts and shape susceptibility to neoplastic development. The convergence between oncogenic risk, progressive renal injury, and metabolic heterogeneity underscores the need to interpret these processes in an integrated way to understand the effects in terrestrial organisms and to establish prevention, diagnostic and monitoring approaches consistent with the severity and dynamics of this toxicity.

Scientific literature consistently emphasizes the need for proactive medical surveillance of high‐risk groups and the monitoring of specific biomarkers of exposure, such as AA–DNA adducts and urinary microRNA alterations. Regulatory restrictions on *Aristolochia*‐based herbal medicines, alongside the development of public awareness campaigns and health policies, are strongly recommended. Despite national and international prohibitions, these exposures continue to occur frequently and remain underdiagnosed worldwide. The persistence of such cases contributes to the ongoing incidence of chronic renal failure and urinary tract cancers, particularly within communities with limited access to health information or specialized medical care (Luciano and Perazella 2014; Marcus and Groll 2015; Zhang et al. 2023).

Environmental contamination by AAs and aristoloxazines forms a critical backdrop for understanding the public health challenges outlined above, since these compounds are now recognized not only as toxic constituents of herbal medicines but also as pervasive pollutants in agricultural soils and rural air. The environmental spread of nephrotoxic, genotoxic, and carcinogenic AAs and AXs has been documented extensively, as Chin et al. (2024) demonstrated that both classes of compounds contaminate more than 400 soil samples from Aristolochiaceae cultivation fields, with aristoloxazine C present in 318 of 320 samples at concentrations reaching 2.8 mg/kg, inhibiting plant growth and reducing soil microbial populations. This contamination is not confined to endemic regions, as Draghia et al. (2021) detected AA‐I in soils and vegetables from non‐endemic gardens, indicating that the natural decay of *Aristolochia clematitis* releases AAs into the soil and contaminates food crops, thereby reinforcing that environmental exposure is far broader than previously assumed. Moreover, Zhang, Chan, and Chan (2022) showed that vegetables such as lettuce, celery, and tomato absorb AAs through their roots and partially translocate them to aerial tissues, while Chan et al. (2016) demonstrated contamination of wheat and corn grown in Balkan soils, confirming that root uptake is a central pathway in the etiology of endemic nephropathy. Beyond ingestion, Au et al. (2025) revealed that humans are also exposed through inhalation, as AA‐I and AL‐I were detected in honey, in masks worn by rural residents and on outdoor surfaces, originating from pollen and from the combustion of *A. clematitis* mixed with wheat remnants for cooking, heating, and fertilizer production; this airborne route, highlighted by recent WHO alerts, represents a previously overlooked yet significant driver of BEN and respiratory disease. Taken together, these findings show that soils, crops, and the atmosphere in regions colonized by AA‐producing species form a multifactorial exposure landscape, where plant decay, root uptake, ecotoxic AXs, and particle‐bound emissions converge to generate a contaminated and highly carcinogenic environment, thereby reinforcing the urgent need for coordinated environmental monitoring, agricultural mitigation, and the regulation of cultural practices that inadvertently amplify human exposure.

#### 2.7.1 Toxicity of Aristolactams as the Invisible Driving Force Behind Human Exposure to Aristolochic Acid

The understanding of aristolactams as active metabolites of AAs has advanced substantially and now reveals a complex toxicological landscape in which bioaccumulation, DNA adduct formation, and interactions with essential cellular pathways converge to explain their profound impact on human health (Figure 2c). Aristolactam I (ALI), in particular, has emerged as a metabolite with extraordinarily high accumulation levels compared with AAI, as shown by Au et al. (2025), who demonstrated that ALI can reach intracellular concentrations up to 900‐fold higher in human renal cells. This intensified accumulation indicates that aristolactam toxicity does not arise solely from the direct action of AAs but rather from the prolonged intracellular persistence of their metabolites, which remain active for extended periods and participate in high‐risk molecular reactions. The extended retention of ALI in renal tissues therefore suggests a central role for this metabolite in the progression of AA‐associated nephropathy.

The rapid uptake of ALI and its selective mitochondrial accumulation, as demonstrated by Zhou et al. (2024), adds another critical layer to the toxicological discussion. Transporter‐independent mitochondrial internalization suggests an unusual and highly permissive mechanism through which ALI directly interacts with structures essential for energetic homeostasis and redox control. Because mitochondrial dysfunction is closely linked to excessive production of ROS, the capacity of ALI to induce oxidative stress and glutathione depletion, reported by Au et al. (2025), reinforces its role not only as a genotoxic agent but also as a potent promoter of systemic cellular injury. This combination of bioaccumulation and mitochondrial disruption makes aristolactams particularly harmful in organs with high metabolic demand, such as the kidney and liver.

A critical aspect of aristolactam toxicity is their strong ability to form DNA adducts, especially the 7‐(deoxyadenosine‐N6‐yl) aristolactam I adduct (dA‐AL‐I) described by Wang, Zhang, et al. (2022) and Wang, Bai, et al. (2022). These adducts are persistent, resistant to repair and remain detectable decades after exposure, as indicated by Reddy et al. (2023). Because such adducts generate characteristic mutations that give rise to Signature 22, which is internationally recognized as the mutational hallmark of AAs exposure, their presence establishes a direct mechanistic connection between aristolactams and the development of upper urinary tract urothelial carcinoma, hepatocellular carcinoma and potentially other epithelial tumors. The long‐term persistence of dA‐AL‐I lesions suggests a sustained risk in which even remote exposures can lead to late mutational events.

Recent literature summarized by Au et al. (2025) also highlights the importance of environmental and dietary exposure routes to aristolactams. Their findings show that the burning of *Aristolochia* species releases AAs into the environment, contaminating agricultural soils and increasing unintentional dietary exposure (Figure 2d). In addition, Au et al. (2023) demonstrated that certain nutrients enhance the formation of ALI–DNA adducts, indicating that diet acts as a critical modulator of aristolactam‐induced carcinogenesis. This means that populations exposed through environmental or dietary pathways may experience amplified risk due to specific nutritional patterns, creating a multifactorial scenario that links contamination, metabolic susceptibility, and cultural habits. Such complexity reinforces the need for public health strategies targeting both environmental mitigation and nutritional education.

Taken together, the findings of Au et al. (2025), Zhou et al. (2024) and Wang, Bai, et al. (2022) present a clear picture: aristolactams are not merely by‐products of AAs but fully active toxic agents capable of inducing extreme bioaccumulation, mitochondrial damage, oxidative stress, persistent DNA adduct formation, and highly characteristic carcinogenic mutations. Their impact on human health is substantial, encompassing chronic nephrotoxicity, progressive fibrosis, renal and hepatic carcinogenesis, and often invisible environmental exposure. Thus, recognizing aristolactams as key drivers of AA‐associated toxicity highlights the urgency of stricter regulatory policies, systematic environmental monitoring, and preventive strategies based on nutritional education and control of contamination sources.

Integrating evidence of aristolactam toxicity with recent advances in spatial multiomics applied to AA‐induced nephropathy reveals a unified framework in which early molecular damage induced by metabolites such as ALI converges with the spatial organization of the kidney to determine the severity and clinical evolution of AAN. Because aristolactams display pronounced bioaccumulation and form persistent DNA adducts, as shown by Au et al. (2023), Wang, Bai, et al. (2022), and Sidorenko et al. (2020), their effects are not distributed uniformly throughout renal tissue. Cortical regions, already known to be particularly vulnerable to proximal tubular cell loss and immune infiltration according to Chen et al. (2022), become critical sites where genotoxic adducts combine with inflammatory microenvironments to accelerate apoptosis and impair regenerative capacity. That means that the spatial heterogeneity described by Chen et al. (2024) is not merely a reflection of renal anatomy but a direct manifestation of differential metabolite distribution and its interaction with intercellular communication pathways such as MHC‐I and CCL, amplifying the vulnerability of specific nephron segments.

Since ALI disrupts mitochondrial function and promotes oxidative stress, its presence in the fibrotic and immune microenvironments identified by spatial multiomics contributes to a self‐sustaining pathological cycle in which inflammation recruits immune cells that further potentiate tubular damage. Activation of M1 macrophages and cytotoxic T cells, as observed by Chen et al. (2022), becomes especially detrimental when occurring in a molecular context already compromised by persistent DNA adducts and dysregulated metabolic pathways, indicating that aristolactams not only initiate injury but also shape the microenvironment to perpetuate and accelerate fibrotic progression. This understanding also explains why AAN evolves rapidly into chronic kidney disease and carries a high risk of urothelial carcinoma, since the continuous presence of mutagenic adducts within inflammatory microenvironments facilitates the selection of clones bearing mutations consistent with Signature 22.

The metabolic reprogramming documented by Li et al. (2024) fits directly into the framework of aristolactam‐induced toxicity, which interferes with mitochondrial processes and depletes cellular antioxidants. Once key enzymes of the Krebs cycle and aerobic respiration are inhibited, as demonstrated by Zhou et al. (2024), cells enter a compromised survival state marked by increased reliance on glycolytic pathways, reduced oxidative phosphorylation and heightened susceptibility to apoptosis. Spatial multiomics show that these metabolic patterns are not uniformly distributed but rather organized in specific regions of the nephron, indicating that renal architecture itself modulates how chemical injury manifests. The presence of distinct metabolic signatures among nephron segments therefore reinforces that aristolactam toxicity is driven not only by intrinsic molecular properties but also by the spatial context of each cell within renal tissue. This integrated perspective suggests that effective therapeutic interventions will need to address not only the elimination of AAs exposure but also the correction of region‐specific metabolic dysregulation and the interruption of inflammatory circuits sustained by aristolactams.

Integrating the molecular toxicity of aristolactams with spatial multiomic insights into AA‐induced nephropathy ultimately reveals a scenario in which the global public health implications become unavoidable, once the extreme bioaccumulation of ALI, the persistent formation of mutagenic adducts and the spatial remodeling of the kidney converge to explain not only the rapid progression of AAN but also its strong association with malignancies such as urothelial and hepatocellular carcinoma. In this context, the spatial understanding of renal responses, including inflammatory and fibrotic microenvironments and segment‐specific metabolic reprogramming, indicates that the clinical manifestations observed in populations exposed environmentally, occupationally or through diet are the result of a multifactorial interaction between persistent chemical damage, anatomical vulnerability, and metabolic susceptibility. Effective public health strategies will therefore need to integrate environmental surveillance, strict control of contaminated herbal products, risk‐based nutritional education and the development of therapeutic interventions that consider renal spatial heterogeneity and the decades‐long persistence of DNA adducts, highlighting the urgency for global policies that recognize AAN and aristolactams as underestimated yet epidemiologically significant threats.

### 2.8 Regulation and Sociocultural Dilemmas in the Use of *Aristolochia* in Herbal Medicines: Between Tradition and Public Health Protection

#### 2.8.1 Phytochemical and Pharmacological Diversity of *Aristolochia* Species and Its Influence on Therapeutic and Toxic Effects

The phytochemical and pharmacological diversity of *Aristolochia* species plays a fundamental role in various cultures and medical traditions, particularly in Traditional Chinese Medicine and in popular medicinal practices across the Amazon region (Lerma‐Herrera et al. 2022; Mueller et al. 2022) (Figure 2d). The genus encompasses a wide spectrum of secondary metabolites, including AAs, aristolactams, terpenes, and flavonoids, whose chemical profiles vary depending on species, habitat, and environmental conditions (Tian‐Shung et al. 2004; Damu et al. 2003; Chauhan et al. 2024). These compounds have been associated with multiple pharmacological activities, such as anticancer, antioxidant, and antimicrobial effects (Michl et al. 2016). Nevertheless, the presence of nephrotoxic and carcinogenic constituents, particularly AA I and AA II, has raised significant public‐health concerns and led to growing regulatory restrictions and intensified research into detoxification strategies and safety‐enhancing approaches (Okhale et al. 2019; Zhou et al. 2019; Fan et al. 2020).

In many cultural contexts, especially in the Amazon region and East Asia, the traditional use of *Aristolochia* species occurs without full understanding of the potential risks involved (Mueller et al. 2022; Soares et al. 2022). At the same time, modern scientific research has proposed promising strategies to mitigate toxicity, such as bioconversion and alkaline processing, which aim to preserve desirable bioactive properties while reducing toxic potential (Nogueira et al. 2021; Schneidewind et al. 2024). These techniques exemplify the integration between traditional knowledge and contemporary pharmacological and toxicological standards, merging local empirical practices with laboratory validation (Teixeira et al. 2023; Tello and Bravo 2024). Despite significant pharmaceutical potential, the absence of standardized detoxification processes remains a challenge, given the pronounced phytochemical variability among *Aristolochia* species across biomes and cultures (Bourhia et al. 2019; Sharmin et al. 2023). Moreover, addressing the challenges of toxicity and chemical variability is essential to ensure the safe therapeutic use of the genus. Recent advances in metabolomic profiling, bioactivity assays, and region‐specific toxicological assessments have provided integrated parameters showing that understanding phytochemical diversity may facilitate the rational development of new drugs strengthening traditional applications through scientific substantiation (Ji et al. 2020; Liu et al. 2020; Tian et al. 2022; Liu et al. 2024).

#### 2.8.2 Extrapolating Toxicological Data From Animal Models to Humans: Limits, Advances, and Implications for *Aristolochia*‐Based Herbal Medicines

Scientific studies addressing the extrapolation of toxicological data from animal models to humans emphasize critical scientific, technological, and ethical dimensions, underlining the need for caution in translating preclinical findings into human risk assessment. Advances in omics technologies and computational modeling have revolutionized mechanistic understanding and improved interspecies predictive capabilities. Simultaneously, Kaplan et al. (2024) noted that the growing ethical commitment to reducing animal use in research has accelerated the development and validation of *New Approach Methodologies* (NAMs), sparking debates about the predictive validity of animal‐derived data in shaping health‐related decisions.

Substantial evidence indicates that animal models often exhibit limited predictive power for human toxicological outcomes, primarily due to significant physiological, metabolic, and behavioral differences among species (Hickey et al. 2024; Ineichen et al. 2024). Therefore, while animal studies remain valuable for providing reference frameworks in risk assessment, a critical stance must be maintained in interpreting their results, balancing the recognition of experimental insights with the uncertainties inherent to interspecies translation.

In this evolving landscape, the integration of in vitro and in silico approaches occupies a central position in contemporary toxicological research. Numerous studies highlight the potential of organoids, microphysiological systems, and machine‐learning–based models as innovative tools to improve extrapolative accuracy and reduce animal use in the medium to long term. These methods deepen understanding of toxicity mechanisms and expand the analytical toolkit available for safety assessments, enriching human risk estimation with mechanistic precision (Zhang, Wang, Yang, et al. 2022; Cattelani et al. 2024).

Additionally, omics sciences such as transcriptomics, proteomics, and metabolomics are providing substantial contributions to toxicological research by unraveling molecular pathways involved in xenobiotic toxicity (Li et al. 2024; Son et al. 2024). When integrated with computational tools, these approaches enable the identification of molecular biomarkers, advance bioinformatics‐based predictive modeling, and reinforce evidence‐driven frameworks for mechanistic risk evaluation (Son et al. 2024).

Selection of animal models must, in turn, be made cautiously and contextually, taking into account the physiological, metabolic, and genetic traits of each experimental species (Alexander‐White 2024). Such rigor is indispensable to ensure data reliability and cross‐species relevance, requiring a mechanistic understanding of toxicokinetic and toxicodynamic nuances in each organism.

Despite methodological progress, challenges persist most notably those linked to differences in exposure levels, genetic diversity, administration routes, and dose scaling. Strategies such as allometric modeling, physiologically based pharmacokinetic (PBPK) modeling, and consideration of gene–environment interactions are being explored to mitigate these obstacles, though inherent uncertainties remain. Nevertheless, the integration of in vitro and in silico mechanistic data is expected to enhance the translational relevance of preclinical studies and reduce late‐stage failure rates in drug development (Johnson and Emiliem 2012; Alexander‐White 2024).

This paradigm shift underscores the importance of integrating methodological, clinical, and epidemiological perspectives when assessing the risks associated with *Aristolochia* use in humans a topic further explored in the next sections through case reports, epidemic episodes, and public‐health analyses.

#### 2.8.3 Clinical and Epidemiological Evidence of Human Intoxication by *Aristolochia*

Human intoxication caused by *Aristolochia* species and their derivatives represents one of the most alarming examples of disease arising from combined environmental and cultural exposure to natural products. The phenomenon transcends regional boundaries, encompassing devastating clinical outcomes, silent epidemics in endemic regions, and profound implications for public health policy and global healthcare systems (Zhou et al. 2023). International recognition of this issue has emerged from the convergence of case reports, large epidemiological studies, and advances in molecular biology, all of which have established a direct causal link among AAs exposure, chronic nephropathy, and the development of urothelial neoplasms, an association with severe consequences for morbidity and mortality (Oleszczuk et al. 2022; Reinoso et al. 2024; Cui et al. 2025).

The earliest case reports, particularly in Europe, first exposed the toxicological risks of plants containing *Aristolochia* used in herbal medicine. During the 1990s, the so‐called “Belgian weight‐loss herb scandal” involved hundreds of patients who developed end‐stage renal failure after undergoing slimming treatments that contained extracts derived from *Aristolochia* species. This outbreak was soon linked to an abnormally high incidence of upper urinary tract carcinomas (Nortier et al. 2000). These sentinel cases not only highlighted the severity of AA‐related toxicity but also revealed widespread underreporting and the lack of effective regulation of botanical products in several regions.

Subsequent investigations uncovered silent, decades‐long epidemics across southeastern Europe, most notably among families residing in the river valleys of the Balkans, where cases of so‐called “Balkan endemic nephropathy” had long puzzled physicians. Advances in molecular epidemiology demonstrated that this condition was, in fact, a toxic nephropathy caused by chronic dietary ingestion of *Aristolochia* seeds contaminating wheat flour, consumed repeatedly over many years (Stiborová et al. 2016). These epidemiological clusters revealed patterns consistent with environmental and familial exposure and underscored the critical necessity of integrated surveillance systems, as many cases were only identified once patients reached terminal stages of kidney failure.

Clinically, AA‐induced nephropathy is characterized by a slow and insidious onset, progressive and irreversible renal decline, and discrete manifestations such as proteinuria, anaemia, and hypertension. Histopathological analyses show prominent tubulointerstitial fibrosis, tubular atrophy, and in many cases, urothelial dysplasia or malignant transformation (Luciano and Perazella 2014; Zhou et al. 2023). The coexistence of rapidly progressive nephropathy with urothelial cancers is now well documented, with clinical series reporting a 30‐ to 50‐fold increase in the incidence of upper urinary tract carcinoma among individuals chronically exposed to AAs (Wu et al. 2012; Chen et al. 2024).

Epidemiological data continue to demonstrate the global breadth of this problem. In Taiwan, where traditional herbal medicine is widespread, population‐based studies have shown epidemic‐level incidences of renal and urothelial carcinomas attributed to *Aristolochia* exposure accounting for up to 60% of certain tumor subtypes (Wu and Wang 2013). In rural regions of China, environmental factors such as grain contamination and co‐exposure to other nephrotoxic or carcinogenic agents like arsenic have been shown to compound the risk and lethality of the disease (Chen et al. 2024). Additional risk factors include cumulative exposure dose, advanced age, female sex, genetic predisposition, and the presence of comorbid chronic diseases (Lukinich‐Gruia et al. 2024). Family and genetic studies have revealed that polymorphisms in genes related to DNA repair and AAs metabolism may modulate individual susceptibility, suggesting new avenues for personalized medicine in the near future (Zhou et al. 2023).

The late recognition of AAs toxicity led to major public‐health shortcomings in the past, with devastating outcomes in terms of disability, need for dialysis, and premature mortality particularly in areas with limited epidemiological surveillance (Grollman 2012). Current preventive strategies focus on strict control over the use and commercialization of *Aristolochia*‐containing products, intensive public‐awareness campaigns, and investment in laboratory and molecular surveillance, including biomarker screening programs for exposed populations (Yun et al. 2015). Clinical research is also aimed at improving early diagnostic methods and exploring therapeutic interventions, although reversibility remains extremely limited in advanced cases (Cui et al. 2025).

Ultimately, *Aristolochia*‐related intoxication underscores the vulnerability of healthcare systems when traditional practices, cultural persistence, and regulatory failures converge to expose populations to chronic and often silent risks. Consequently, this issue has become a strategic and growing concern in global public health demanding coordinated international responses, sustained epidemiological vigilance, and a dynamic interdisciplinary research agenda linking molecular biology, clinical nephrology, public‐health policy, and cultural studies.

#### 2.8.4 Environmental Exposure to Aristolochic Acids and Its Impact on Public Health and Food Safety: Soil Contamination and the Food Chain

Environmental exposure to AAs currently represents one of the most complex challenges to food safety, environmental integrity, and collective health, with new evidence emerging each year that expands the understanding of the risks associated with these compounds. The long‐standing medicinal use of *Aristolochia* had already placed AAs under suspicion due to their recognized nephrotoxic and carcinogenic potential, especially after their association with Balkan endemic nephropathy and urothelial carcinomas. However, recent literature reveals that environmental exposure, through the persistence of AAs in soil and their silent infiltration into agricultural production chains, may be of comparable or even greater magnitude, calling for urgent attention from regulatory bodies and continued research on mitigation strategies (Li et al. 2018; Faria et al. 2021).

Because of their chemically stable and degradation‐resistant nature, AAs can persist in agricultural soils for months, particularly in acidic environments, effectively creating long‐term environmental reservoirs. This persistence facilitates their uptake by plant roots and accumulation in various food crops, including wheat, maize, tomatoes, and leafy vegetables commonly consumed worldwide (Li et al. 2016; Gruia et al. 2018; Zhang et al. 2024). Experimental evidence confirms not only root absorption but also the translocation of AAs to edible plant parts, firmly establishing contamination throughout the food chain and suggesting possible dietary exposure even in populations distant from *Aristolochia* cultivation areas.

This phenomenon represents a hidden risk to populations that have never deliberately used *Aristolochia*‐based herbal medicines. Epidemiological and analytical studies have detected measurable concentrations of AAs in agricultural products and their derivatives, illuminating the broad extent of the problem. Individuals consuming food from contaminated regions are unknowingly exposed to subclinical doses of these toxic compounds, a scenario that may contribute to increased rates of renal disease and urothelial or hepatic tumors even in regions where *Aristolochia* holds no role in traditional medicine (Jelaković, Castells, et al. 2015; Zhou et al. 2023; Li et al. 2024).

The risk is further compounded by contamination of raw plant materials used in herbal products, which may occur during cultivation without any intentional inclusion of *Aristolochiaceae*. This underscores the urgent need for rigorous revisions in certification, traceability, and labeling protocols, as well as the refinement of laboratory methods for the detection of AAs in phytotherapeutics, food, and beverages (Mogaddam et al. 2020; Silva et al. 2020). These findings draw a crucial link between agricultural soil quality and the safety of plant‐based food products, reinforcing the relevance of environmental assessment as part of food‐quality and public‐health policies.

Recent studies have also highlighted the synergistic potential between AAs and other environmental contaminants. The co‐presence of polycyclic aromatic hydrocarbons, phthalates, and heavy metals has been shown to enhance the carcinogenic potency of AAs by increasing DNA adduct formation (Zhang et al. 2024). Rural and peri‐urban areas, often burdened with higher concentrations of these pollutants, therefore become complex zones of overlapping risk, demanding integrated approaches at the intersection of environmental health and food safety. Beyond the direct implications for human populations, the ecological consequences are equally critical, as AAs disrupt soil microbial communities and affect biogeochemical processes fundamental to long‐term fertility and agricultural productivity (Gruia et al. 2018). This erosion of soil biodiversity increases the vulnerability of local food systems in endemic or contaminated landscapes, although remediation strategies such as phytoremediation remain restricted to controlled or small‐scale applications (Wang et al. 2021).

Supporting this broader environmental context, recent monitoring campaigns in Serbia have demonstrated how AAs and ALs permeate multiple environmental matrices. According to an extensive and recent study by Au et al. (2025), contamination has been identified in honey, airborne pollen, surface dust, and combustion‐derived aerosols. Honey collected from weekend markets in Niš showed detectable levels of AA‐I in three of 42 samples, with concentrations ranging from 222 to 390 pg/g. Analyses of face masks worn downwind from *A. clematitis* flowering areas detected AA‐I in all samples, exhibiting both distance‐ and exposure‐duration–dependent accumulation. Complementary environmental sampling using alcohol swabs in villages and in the city of Niš identified AAI, AAII, AL‐I, and AL‐II in 60, 5, 12, and 3 of 261 samples, respectively, with AAI reaching 2470 pg. near flowering *A. clematitis* . Further investigations revealed that burning wheat remnants mixed with *A. clematitis* released AL‐I into the air, with strong correlations (r > 0.97) between airborne AL‐I and the concentrations of AAI and AL‐I in the burned plant material, and additionally showed that AL‐I was predominantly associated with particulate aerosols. Taken together, these findings illustrate how environmental contamination in the Balkan Peninsula extends beyond soil and crops, permeating air, household surfaces, and locally produced foods, thereby intensifying chronic exposure risks for communities living in endemic regions.

Collectively, these findings emphasize the need for robust public policies devoted to continuous monitoring of agricultural soils and plant‐derived products, alongside the development of advanced analytical technologies for detecting AAs in food, soil, and water matrices. Contemporary interdisciplinary research demonstrates that environmental toxicology, agricultural sustainability, and human health are deeply interconnected. This integrated perspective redefines traditional paradigms in environmental and food toxicology, highlighting the necessity of cohesive preventive strategies, collaborative scientific efforts, and coordinated international policies to anticipate, identify, and mitigate the multifaceted risks posed by AAs contamination.

#### 2.8.5 Strategies for Diagnosis, Monitoring, and Management of *Aristolochia*‐Induced Intoxication

Intoxication caused by *Aristolochia* species primarily due to the presence of AAs constitutes a significant public‐health concern that demands integrated strategies for diagnosis, monitoring, and clinical management. Owing to their well‐documented nephrotoxic and carcinogenic potential, particularly in the genesis of aristolochic acid nephropathy and urothelial malignancies, an effective approach must encompass clinical, laboratory, regulatory, and public health dimensions.

The diagnosis of *Aristolochia*‐related intoxication presents major clinical challenges, largely due to the insidious and nonspecific nature of early renal impairment associated with AAs exposure. Symptoms often appear only at advanced stages, when tubulointerstitial damage and chronic renal insufficiency have already developed. Consequently, detailed anamnesis becomes a cornerstone of clinical assessment. A precise investigation into patients' history of herbal remedy use and possible environmental or occupational exposure in endemic regions is essential for identifying those at risk (Reinoso et al. 2024). Histopathological findings underscore this diagnostic complexity: renal tissue usually shows interstitial fibrosis, tubular atrophy, and, in some, dysplasia or malignant transformation features that become apparent only in later disease stages.

At the laboratory level, high‐performance liquid chromatography (HPLC) remains one of the most reliable methods for detecting and quantifying AAs in biological samples and plant materials. Advances include the development of molecular diagnostic assays such as lateral‐flow immunochromatographic tests, which combine robustness, high sensitivity, and specificity for the identification of *Aristolochia* DNA in suspect samples (Thongkhao et al. 2022; Dugăeşescu and Andrei‐Bitere 2024). The integration of these analytical tools enhances early diagnosis and supports broader surveillance programs, strengthening public health responses to toxic exposure.

Following diagnosis, continuous monitoring becomes critical to mitigate disease progression and associated complications. Pharmacovigilance programs, coordinated by health agencies and national surveillance networks, play an essential role in the early detection of new cases and in tracing contaminated product batches. Several countries have implemented strict regulations prohibiting or restricting the use of *Aristolochia* in herbal preparations (Oleszczuk et al. 2022). Nonetheless, the continued reports of intoxication and the widespread availability of unregulated products, particularly through online markets, demand ongoing refinement of surveillance, and enforcement mechanisms (Zhou et al. 2023).

From a clinical standpoint, patients diagnosed with aristolochic acid nephropathy require long‐term multidisciplinary follow‐up due to the high risk of progression to end‐stage renal disease and associated urinary‐tract malignancies. Optimal management protocols include regular monitoring of renal function through laboratory testing, combined with periodic imaging or cystoscopy to detect early neoplastic alterations, as clinically indicated (Oleszczuk et al. 2022; Cui et al. 2025). Such proactive management aims to identify complications promptly and enable specific interventions that may slow disease progression or treat malignant outcomes at an early stage.

Preventive measures at the public‐health level are equally crucial to interrupt ongoing exposure. International regulatory agencies, including the World Health Organization (WHO), have issued recommendations and restrictions concerning the sale and consumption of *Aristolochia*‐containing products. However, the informal trade and digital distribution channels continue to hinder effective control, highlighting the importance of rigorous enforcement and sustained educational campaigns directed at both healthcare professionals and the general public (Oleszczuk et al. 2022; Tian et al. 2022). Moreover, workers handling potentially contaminated herbal materials including herbalists, agricultural laborers, and industrial processors must be protected through biosafety standards, training in proper handling procedures, and the use of appropriate personal protective equipment (Kwak and Chan 2024).

Despite the strategies already in place, persistent challenges remain. The variability of toxicity among different *Aristolochia* species, the difficulty in tracing exposure routes, and the deep cultural embedding of *Aristolochia* in traditional medicine practices complicate the establishment of comprehensive global control. These factors contribute to substantial underreporting and underdiagnosis, emphasizing the need for continuous investment in research, diagnostic innovation, and novel therapeutic and preventive approaches.

An effective response to *Aristolochia* intoxication thus depends on an integrated effort that combines scientific research, technological innovation, active health‐system coordination, and robust regulatory policies. International cooperation, intersectoral communication, and continuous professional and community education are indispensable for improving detection, monitoring, and clinical management strategies. Ultimately, these synergistic actions are crucial to strengthening public‐health frameworks, protecting vulnerable populations, and safeguarding global food and medicinal safety in the context of AAs exposure.

#### 2.8.6 Main Regulatory Challenges, Sociocultural Dilemmas, and Public Policies Related to the Medicinal Use of *Aristolochia*

The regulation of herbal medicines, particularly those containing *Aristolochia*, a genus associated with severe toxicological risks, reveals a global landscape marked by tensions between public health protection and the preservation of cultural traditions. This dilemma is especially pronounced in regions where traditional medicine remains deeply embedded within the sociocultural fabric, functioning not only as a therapeutic system but also as a component of collective heritage transmitted across generations. In many of these settings, particularly in West Africa, Southeast Asia, and Mesoamerica, the absence of harmonized regulatory frameworks results in heterogeneous and frequently insufficient approaches to ensuring the safety and efficacy of *Aristolochia*‐based preparations. Major herbal markets often operate under non‐binding guidelines with minimal requirements for toxicological testing or clinical validation, perpetuating population vulnerability to contamination and the adverse effects of AAs (Sangho et al. 2023; Clay 2024).

The lack of effective regulation contributes to misinformation and the circulation of potentially hazardous products, as seen in Ghana, where herbal medicines containing *Aristolochia* remain widely accessible despite recurrent reports of toxicity. In recent years, several countries have attempted to integrate traditional healing systems with formal healthcare structures, as observed in Nigeria, where initiatives aim to align quality and safety standards without delegitimizing indigenous knowledge. However, such efforts encounter both practical and symbolic challenges, as traditional healers and local communities often perceive external regulatory frameworks as threats to cultural autonomy and the authenticity of ancestral practices, as highlighted by Geck et al. (2020) and Eruaga et al. (2024).

The cultural and spiritual significance of herbal therapies extends beyond pharmacological utility, making them essential health resources in rural and underserved regions where access to formal medical care is limited. This sociocultural embeddedness helps explain the reluctance of many practitioners and communities to subject traditional remedies to biomedical evaluation, generating persistent tension between tradition and modernity. In Mesoamerican contexts, resistance to adopting contemporary regulatory standards illustrates the disconnect between biomedical structures and local healing knowledge, underscoring the need for culturally sensitive and participatory approaches (Gupta et al. 2023).

In response to these complexities, policymakers have begun examining models that integrate scientific regulation with respect for traditional health practices. The European regulatory framework offers a noteworthy example by allowing exemptions for traditional herbal medicines with a well‐documented history of use while still requiring evidence of manufacturing quality and safety monitoring. This approach demonstrates how public policy can accommodate cultural diversity without compromising public health responsibilities. Its success, however, depends on sustained dialogue among regulatory authorities, healthcare professionals, and custodians of traditional knowledge, fostering mutual trust and shared responsibility for the safe use of *Aristolochia* and other medicinal plants (Cloatre and Urquiza Haas 2020).

The differences between regulatory policies in the European Union, China, and West Africa reveal contrasting levels of prohibition, analytical standards, and oversight related to the use of *Aristolochia* and products containing AAs, reflecting distinct historical and sociocultural contexts that shape regulatory harmonization. In the European Union, strict regulations emerged after well‐documented toxicity incidents, such as the Belgian slimming‐clinic case described by Gilbert (2011), which prompted the formal prohibition of *Aristolochia* by the European Medicines Agency and its exclusion from the BELFRIT list, as analyzed by Maggini et al. (2018). The analytical standards adopted in the region, based on techniques such as liquid chromatography and mass spectrometry, demonstrate high precision for detecting AAs and support systematic and effective surveillance, as shown by Martena et al. (2007). This model contrasts sharply with that of China, where the historical use of *Aristolochia* within traditional Chinese medicine poses regulatory challenges even after the removal of several species from the Chinese Pharmacopoeia by the CFDA, as reported by Tian et al. (2022) and Gao et al. (2017). Testing standards remain less rigorous, and there is an ongoing need for systematic assessments of toxicity and carcinogenicity (Li et al. 2020). In West Africa, especially in Nigeria, there is a widespread absence of binding regulations, standardized labeling, and analytical testing, despite the continued circulation of products containing AAs, reinforcing a public health vulnerability shaped by reliance on traditional medicine and limited state regulatory capacity (Okhale et al. 2019).

The comparison of these regulatory systems reveals that, while the European Union maintains broad prohibitions and strong inspection mechanisms, China and West Africa contend with regulatory gaps shaped by the intersection of traditional practice and limited institutional infrastructure. Harmonizing regulatory policies could be strengthened by incorporating European standards for analytical testing and surveillance, while respecting the cultural and operational characteristics of Chinese and West African healthcare systems. Achieving this requires enhanced phytochemical characterization, improved monitoring standards, and communication strategies that reduce the circulation of unsafe products. Effective regulatory integration will depend on the adoption of shared minimum requirements, such as quantifiable limits of toxic compounds, adequate labeling, and comparable analytical methods, enabling more consistent and equitable safety control for products containing AAs. This integrated approach provides a solid foundation for reducing health risks while supporting policies that preserve traditional practices without compromising collective well‐being.

Within this broader context, it is important to highlight how the widespread belief in the inherent harmlessness of “natural” products can obscure severe health risks. The common misconception that plant‐derived substances are automatically safe underscores the importance of rigorous evaluation and educational initiatives to minimize public vulnerability. Integrating awareness campaigns with regulatory strategies is therefore essential to counteract this misconception and protect public health while respecting traditional medicinal knowledge.

## 3 Conclusion

The conclusion has been expanded substantially to provide a more comprehensive synthesis of the main findings, their implications for public health and regulatory policies and clearer directions for future research. We appreciate this recommendation, as the strengthened conclusion now offers a more meaningful, integrative and scholarly closing to the manuscript. Taken together, the toxicological, ecological, and epidemiological evidence reviewed here demonstrates unequivocally that AAs and their metabolites, particularly aristolactams, represent a persistent global public health threat that transcends their traditional medicinal use.

The historical reliance on *Aristolochia* species, combined with gaps in regulatory enforcement and widespread unawareness of the risks, has enabled continuous human exposure through medicinal preparations, contaminated herbal products, environmental reservoirs, food‐chain transfer, and airborne particles generated through biomass burning. This complex exposure landscape underscores the urgent need for harmonized international regulations that explicitly prohibit the medical or commercial use of *Aristolochia* species, expand surveillance of marketed herbal formulations and enforce accurate botanical identification throughout the supply chain. Strengthening biomonitoring capacities is essential, particularly as advances in molecular toxicology and multi‐omics have begun to reveal more sensitive and specific biomarkers of exposure, allowing earlier detection of DNA adduct formation, tissue damage, and long‐term carcinogenic risk. Environmental management strategies also require prioritization as recent findings reveal that contaminated soils, crop uptake and atmospheric dissemination contribute to a sustained cycle of human environmental exposure that is often overlooked by public health frameworks.

Investment in remediation technologies, ecological monitoring and risk communication strategies will be critical to interrupt these pathways. From a scientific perspective, future research should deepen our understanding of the metabolic activation and bioaccumulation of AAs and aristolactams, their population‐level exposure patterns and their contribution to the global burden of kidney disease and urothelial cancers. Ultimately, protecting public health requires a coordinated effort that reconciles ethnobotanical traditions with contemporary toxicological evidence, ensuring that cultural practices are preserved without compromising safety. Achieving this balance will depend on regulatory vigilance, public education, improved diagnostic tools and the replacement of *Aristolochia*‐based preparations with safe, evidence‐based alternatives, enabling the global community to mitigate the persistent risks associated with AAs exposure.

## Author Contributions

**Victor Ventura de Souza:** writing – original draft, investigation, formal analysis, conceptualization. **Lara Rodrigues De Andrade:** writing – formal analysis, conceptualization. **Tatiana da Silva Souza:** writing – review and editing – original draft, supervision, formal analysis, conceptualization.

## Funding

The authors have nothing to report.

## Conflicts of Interest

The authors declare no conflicts of interest.

## Data Availability

Data sharing not applicable to this article as no datasets were generated or analysed during the current study.
